# Short hydrogen bonds enhance nonaromatic protein-related fluorescence

**DOI:** 10.1073/pnas.2020389118

**Published:** 2021-05-17

**Authors:** Amberley D. Stephens, Muhammad Nawaz Qaisrani, Michael T. Ruggiero, Gonzalo Díaz Mirón, Uriel N. Morzan, Mariano C. González Lebrero, Saul T. E. Jones, Emiliano Poli, Andrew D. Bond, Philippa J. Woodhams, Elyse M. Kleist, Luca Grisanti, Ralph Gebauer, J. Axel Zeitler, Dan Credgington, Ali Hassanali, Gabriele S. Kaminski Schierle

**Affiliations:** ^a^Chemical Engineering and Biotechnology, University of Cambridge, Cambridge CB3 0AS, United Kingdom;; ^b^Condensed Matter and Statistical Physics, The Abdus Salam International Centre for Theoretical Physics, 34151 Trieste, Italy;; ^c^International School for Advanced Studies (SISSA), 34136 Trieste, Italy;; ^d^Institut für Physik, Johannes Gutenberg Universität, 55128 Mainz, Germany;; ^e^Department of Chemistry, University of Vermont, Burlington, VT 05405;; ^f^Departamento de Química Inorgánica, Analítica y Química Física, Instituto de Química Física de los Materiales, Medio Ambiente y Energía, Facultad de Ciencias Exactas y Naturales, Universidad de Buenos Aires, C1428EHA Buenos Aires, Argentina;; ^g^Cavendish Laboratory, University of Cambridge, Cambridge CB3 0HE, United Kingdom;; ^h^Department of Chemistry, University of Cambridge, Cambridge CB2 1EW, United Kingdom;; ^i^Division of Theoretical Physics, Ruđer Bošković Institute, 10000 Zagreb, Croatia

**Keywords:** short hydrogen bond, intrinsic fluorescence, ultraviolet fluorescence

## Abstract

Fluorescence in biological systems is usually associated with the presence of aromatic groups. Here, by employing a combined experimental and computational approach, we show that specific hydrogen bond networks can significantly affect fluorescence. In particular, we reveal that the single amino acid L-glutamine, by undergoing a chemical transformation leading to the formation of a short hydrogen bond, displays optical properties that are significantly enhanced compared with L-glutamine itself. Ab initio molecular dynamics simulations highlight that these short hydrogen bonds prevent the appearance of a conical intersection between the excited and the ground states and thereby significantly decrease nonradiative transition probabilities. Our findings open the door to the design of new photoactive materials with biophotonic applications.

## Significance

Intrinsic fluorescence of nonaromatic amino acids is a puzzling phenomenon with an enormous potential in biophotonic applications. The physical origins of this effect, however, remain elusive. Herein, we demonstrate how specific hydrogen bond networks can modulate fluorescence. We highlight the key role played by short hydrogen bonds, present in the protein structure, on the ensuing fluorescence. We provide detailed experimental and molecular evidence to explain these unusual nonaromatic optical properties. Our findings should benefit the design of novel optically active biomaterials for applications in biosensing and imaging.

Short peptides void of any aromatic residues have been shown to display an intrinsic fluorescence in the visible range (1, 2). This has primarily been observed in fibrillar protein structures linked to neurodegenerative diseases, such as Alzheimer’s, Parkinson’s, and Huntington’s diseases (3–6). Furthermore, optical properties of double amino acid–based nanowires have also been reported, consisting either of two nonaromatic or two aromatic amino acids (2, 7, 8). We have previously suggested that the fluorescence of nonaromatic short crystal structures forming part of the amyloid-β protein is enhanced by proton delocalization (5). We have hypothesized that one of the prerequisites for this fluorescence observed in either amyloid structures or short peptide nanowires is related to hydrogen bonding or aromatic interlocks, which, for the latter, decreases the bandgaps down to the semiconductive regions (9).

Despite our previous suggestion that proton delocalization is strongly coupled to this intrinsic fluorescence, its direct role on putative fluorescing states has not been elucidated. We have therefore searched for a model system, such as a single amino acid–based structure, that displays similar optical properties to amyloid fibrils and is permissive to more sophisticated computational approaches. We have been inspired by the small peptide nanostructures that have been pioneered by the Gazit laboratory (9) and by the fact that there are several neurodegenerative diseases that have been connected with an increased level of glutamines produced as part of a protein, as for example, Huntingtin in Huntington’s disease, which renders the protein more aggregation prone (10). It has been known that the amide group in L-glutamine (L-glu) is highly labile and therefore can rapidly hydrolyze. We show here that the single amino acid L-glu, upon heating in water, can form a nanostructural material with optical properties similar to the ones observed in other amyloid fibrils such as in fibrils of amyloid-beta, alpha-synuclein, or tau (4, 11, 12).

Using X-ray diffraction (XRD), we show that L-glu dissolved in water and, upon heating, becomes cyclized, forming a previously unreported structure which resembles L-pyroglutamine and which has been reported to be a component of amyloid-β in the brain (13) but involves a low-barrier hydrogen-bonded anionic dimer with an ammonium counterion. We have termed the structure, that is, L-pyroglutamine complexed with an ammonium ion, L-pyro-amm. L-pyro-amm has a microcrystalline plate morphology as shown by scanning electron microscopy (SEM). The newly formed solid has a unique hydrogen bond (HB) network formed by very strong hydrogen bonds (SHB) with a length of ∼2.45 Å, which is confirmed by using terahertz time-domain spectroscopy (THz-TDS).

By employing a combination of electronic structure calculations and ab initio molecular dynamics in the ground and excited state, we provide an interpretation of the reported experiments. We illustrate the important contribution of different vibrational distortions on the optical properties. Furthermore, our simulations identify the origins of the nonaromatic fluorescence in L-pyro-amm, demonstrating the key role played by the SHBs, which prevent the appearance of a conical intersection, significantly reducing the chances of nonradiative relaxation toward the ground state and hence increasing the likelihood of fluorescence to occur.

## Materials and Methods

### Experimental.

#### Sample preparation of L-glutamine.

L-glu (#G3126, #G8540, Sigma-Aldrich) and L-pyroglutamine (L-pyro) (#83160, Sigma-Aldrich) were dissolved in 18.2 Ω MilliQ H_2_O at a concentration of 0.3 M or 1 M. Aliquots were placed in a 65 °C oven since heating up proteins to 65 °C increases the formation of amyloid structures as reported previously (14). Each aliquot was rotated to dissolve the powder once a day. Samples were either analyzed in liquid form or dried on a glass or quartz coverslip (#043210.KG, Alfa Aesar) either at room temperature (RT) or on a heat block set to 50 °C. L-pyro-amm formed translucent crystals when dried.

#### Emission and excitation wavelength scans.

Emission and excitation spectra were taken on a Hitachi F-4500 Fluorescence Spectrophotometer (Hitachi High-Technologies Corporation) at RT in a quartz cuvette. For measurements, the excitation slit resolution was or 10 nm, and the emission slit resolution was 20 nm. The photomultiplier tube voltage was set at 950 V, and the scan speed was set at 240 nm/min. The excitation scan was measured between 250 to 400 nm, and the emission filter was set to the emission maxima of the sample stated in the figure legend, with a slit resolution of 20 nm. The emission scan was measured between 380 to 560 nm, and the excitation filter was set to the excitation maxima of the sample stated in the figure legend using a slit resolution of 5 nm. Four measurements were taken for each sample, which were repeated at least three times, and the background (air or H_2_O) was subtracted from the average.

#### Absorption measurements.

Absorption measurements were taken on a UV-Vis-NIR Spectrophotometer, UV-3600 Plus (Shimadzu) and Cary 6000i (Agilent). L-glu, L-pyro, or L-pyro-amm solutions, 1 M or 0.3 M, were measured in 10 mm QX cuvettes (Hellma Analytics) or dried on quartz coverslips. Measurements were taken between wavelengths 200 to 800 nm using 1 nm steps at a slow scan speed and a 1 nm resolution. The light source change wavelength was set at 393 nm, and the grating change wavelength was set at 750 nm. Both direct and integrating sphere measurements were taken and showed little difference in results; direct measurements are shown in the manuscript. Samples were measured at least three times, and the experiments were repeated at least three times; measurements were then averaged and H_2_O or coverslip only control was subtracted.

#### SEM.

SEM was performed using a FEI Magellan 400 HR SEM at an acceleration voltage of 2 kV. L-pyro-amm samples were lyophilized by freezing in liquid nitrogen and freeze drying in a LyoQuest −85 (Telstar), and then they were imaged on a glass coverslip.

#### XRD.

L-pyro-amm was dried on a glass coverslip in a 50 °C oven and then at RT until crystals formed. Single-crystal XRD (SCXRD) measurements were performed at 180 K with a Bruker D8 QUEST PHOTON 100 diffractometer, which utilized a Cu Kα radiation (λ = 1.54 Å) and an APEX II CCD. Absorption corrections were made using SDABS, and data integration and reduction were performed with SAINT+. All nonhydrogen atoms were refined isotropically and anisotropically followed by the inclusion of the hydrogen atoms (determined using the excess electron density) and refinement isotropically. The structure is deposited in The Cambridge Crystallographic Data Centre (no. 1981551).

#### THz*-*TDS.

All THz-TDS spectra were acquired using a commercial TeraPulse 4000 Spectrometer (TeraView Ltd). Samples were prepared for THz-TDS measurements by diluting the solid air-dried L-pyro-amm with polyethylene (∼10% weight/weight concentration) by gentle mixing using an agate mortar followed by pressing into 2 mm thick, 13 mm diameter pellets using a hydraulic press. All THz-TDS spectra shown are a result of the division of sample and blank datasets, with the blank dataset representing the THz-TDS response of a pellet of pure polyethylene.

### Theoretical.

#### Density*-*functional theory*-*THz calculations.

Calculations were performed using both the CRYSTAL17 (15) and Quantum ESPRESSO (16) software packages. Geometry optimizations and vibrational analyses performed with the CRYSTAL17 code utilized the atom-centered split-valence triple-zeta 6-311g(2d,2p) basis set for all atom types. Based on a previous study related to ionic molecular crystals (17), the range-corrected WB97-X (18) functional was used. The vibrational analysis was performed within harmonic approximation, and infrared intensities were determined using the Berry phase method (19). Energy convergence criteria were set to ∆E < 10^−8^ and 10^−11^ hartree for the geometry and frequency calculations, respectively.

#### Periodic time*-*dependent density*-*functional theory excited*-*state calculations.

Simulations were performed using the fully periodic Quantum ESPRESSO software package. The Becke, three parameter, Lee–Yang–Parr (B3LYP) hybrid density functional was used with an energy cutoff of 40 Ry. The calculations of the excited state were performed within the framework of time-dependent density-functional theory (TDDFT) using the Liouville–Lanczos formalism implemented in the freely available Quantum ESPRESSO package (20). In this approach, the optical spectra are obtained directly over the wide spectral range without taking into account the numerically complex calculations of the single exited states. We used a plane-wave basis set, and the electron–ion interactions were taken into account via norm-conserving Martins–Troullier pseudopotentials (21). To determine the ground-state wave function, we used the gamma point of the Brillouin zone. All the periodic calculations employed the computationally demanding B3LYP (22) hybrid functional, and the kinetic energy cutoff of 40 Ry was used for the wave functions. The intrinsic bandwidth for the spectra was set to 0.003 Ry (∼0.0408 eV).

#### Periodic structure geometry optimization.

The structures obtained from the experiments were first geometrically optimized at 0 K using the Broyden–Fletcher–Goldfarb–Shanno minimization algorithm implemented in the CP2K (23, 24) package. A convergence criterion for the wave function optimization was used as 5 × 10^−7^ au. Applying the method of the Gaussian and plane wave, the wave function was expended in the Gaussian double-zeta valence polarized basis set. The cutoff for the electronic density was set to 300 Ry. We used the gradient correction to the local density approximation, and the core electrons were treated via Goedecker–Teter–Hutter pseudopotentials (25). In all the calculations, we used the Becke–Lee–Yang–Parr (26) functional with the D3(0) Grimme (27) dispersion corrections for the Van der Waals interactions.

#### Ab initio molecular dynamics simulations.

Ab initio molecular dynamics simulations (AIMD) were performed using the Quickstep algorithm implemented in CP2K. In these calculations, the propagation of the nuclei was taken into account within the framework of the Born–Oppenheimer approximation. The simulations were performed in the canonical ensemble, and the temperature was controlled during the simulations by using the velocity-rescaling thermostat (28). We used the time step of 0.5 femtoseconds to update the nuclear coordinates and velocities while the total length of the simulations for each system was 50 picoseconds.

#### Excited*-*state cluster calculations.

A set of excited-state calculations was performed on glutamine clusters in order to understand the role of the environment on the optical properties. Specifically, the optical properties of L-pyro-amm were investigated using various isolated cluster models with the Gaussian09 (29) software package. The clusters were extracted directly from the crystal structure and used in various combinations (dimers, trimers, tetramers) to perform TDDFT calculations. A split-valence triple-zeta 6-311g(2d,2p) basis set was used for all atom types together with the hybrid B3LYP functional. Some benchmark simulations, comparing the optical properties obtained from the periodic calculations using B3LYP to range-corrected hybrid functionals like the Coulomb-attenuating method (CAM)-B3LYP, were also performed with these clusters.

We also performed a series of excited-state optimizations on various model systems built from L-pyro-amm in order to examine the nature of the geometrical distortions that occur on the lowest electronic excited state. These calculations were also performed with the Gaussian09 software package. All clusters were surrounded with a continuum dielectric constant of 80, representing pure H_2_O. The 6-311G(2d,2p) basis set was used for all atoms together with the range-corrected hybrid functional CAM-B3LYP (30). The clusters were first optimized in the ground state after which they were optimized on the first electronic excited state.

#### Nonadiabatic decay probabilities using excited*-*state molecular dynamics.

Excited-state AIMD was employed, as implemented in the LIO quantum-chemical package (https://github.com/MALBECC/lio) (31–34), to analyze the influence of three key factors determining the optical properties of L-pyro-amm: 1) the formation of a SHB, 2) the presence of ammonium, and 3) the combination of ring deplanarization and carbonyl stretching. Our model system for this study was the L-pyro-amm dimer with a single SHB. In order to assess the influence of the ammonium ion on the optical properties of L-pyro-amm, AIMD was performed on both the L-pyro-amm with and without the ammonium ion. Analogously, in order to shed light on the role of the SHB in L-pyro-amm fluorescence, and in addition to performing simulations of L-pyro at the natural SHB distance of 2.5 Å, we performed three sets of simulations of L-pyro constraining the HB distance to 3.0, 3.5, and 4.5 Å, respectively. For each one of the simulations mentioned before, five replicas were taken.

The initial structures for this analysis were extracted from the ground-state AIMD described above. Subsequently, 3 ps of ground-state AIMD at 300 K was performed to equilibrate the system and was followed by 1 ps of excited-state AIMD. The $S_{1}\to S_{0}$ nonradiative transition probability ($NRP_{S_{1}\to S_{0}}$) was computed for every time step using the TDDFT-based trajectory surface hopping algorithm without permitting decays to the ground state (35–37). For further detail on this implementation, please refer to ref. 38. The nonadiabatic coupling elements between S0 and S1 (Eq. 1) can be estimated analytically by employing the method introduced by Tapavicza et al. (39).

$$\sigma_{S0,S1}=\langle {\text{Ψ}}_{S0}\left(r,R\left(t\right)\right)\text{|}\nabla_{R}{\text{|Ψ}}_{S1}\left(r,R\left(t\right)\right)\rangle \frac{dR}{dt},$$ [1]

where R represents the nuclear geometry, and r represents the electronic coordinates, and ${\text{Ψ}}_{S0}$ and ${\text{Ψ}}_{S1}$ are the electronic wave functions on the ground and excited state, respectively. In this context, $NRP_{S_{1}\to S_{0}}$ is expressed as the following:

$$NRP_{S_{1}\to S_{0}}\;\left(t\right)=-2\overset{t+\Delta t}{\underset{t}{\int }}dt^{'}\frac{Re\left[C_{S1}C_{S0}^{*}\sigma_{S0,S1}\left(t^{'}\right)\right]}{C_{S0}C_{S0}^{*}}\;,$$ [2]

where C coefficients satisfy

$$i\hbar \frac{\partial C_{i}\left(t\right)}{\partial t}=C_{i}\left(t\right)\omega_{i}-i\hbar \sum_{j=0}^{N_{states}}C_{j}\sigma_{ij},$$ [3]

and $\omega_{i}$ are the TDDFT excitation energies. Hence, the total nonradiative decay probability at time *t*, $P{\left(S_{1}\to S_{0}\right)}_{t}$, is a function of $NRP_{S_{1}\to S_{0}}\left(t\right)$ and the population of $S_{1}$:

$$\;\;\;\;\;\;\;\;\;\;\;\;\;\;\;\;\;\;\;P{\left(S_{1}\to S_{0}\right)}_{t}=\left(1-\overset{t}{\underset{0}{\int }}dt^{'}P{\left(S_{1}\to S_{0}\right)}_{t^{'}}\right)\;NRP_{S_{1}\to S_{0}}\left(t\right),$$ [4]

where $1-\int_{0}^{t}dt^{'}P{\left(S_{1}\to S_{0}\right)}_{t^{'}}$ is the population of $S_{1}$. Finally, the accumulated nonradiative decay probability in the interval {0, t} is

$$ANRP\left(t\right)=\overset{t}{\underset{0}{\int }}dt^{'}P{\left(S_{1}\to S_{0}\right)}_{t^{'}}.$$ [5]

It is important to note that in order to enable the estimation of $NRP_{S_{1}\to S_{0}}\left(t\right)$ at all times, the system must remain in the $S_{1}$ state during the entire simulation, and therefore in the calculations presented here, the switches between surfaces are avoided. As mentioned above, this methodology was applied to five different initial conditions of the dimer at HB distances of 3.0, 3.5, and 4.5 Å, and to the dimer-amm at SHB distance. In order to avoid introducing large structural deformations, the distance constraints in the HB were introduced in combination with position restraints on the nitrogen and the $C_{\beta }$ of the pyroglutamic ring.

The excited-state AIMDs were performed employing the density-functional theory (DFT) using the hybrid functional PBE0 level (40) with a 6-31G basis set and a time step of 0.5 fs. For the computation of excitation energies, excited-state gradients, and nonadiabatic coupling vectors, the linear response TDDFT method was used within the Tamm–Dancoff approximation (39, 41).

## Results and Discussion

It has long been known that polyglutamine can form amyloid-like fibrillar structures in vitro. The more glutamine residues in the polyglutamine polymer, the faster the aggregation propensity of the polypeptide chain. This led us to investigate whether L-glu on its own, under conditions which normally promote fibril formation, such as an increase in temperature (14), was able to form structures with similar optical properties as recently observed for amyloid fibrils (5, 42, 43).

**Fig. 1.**
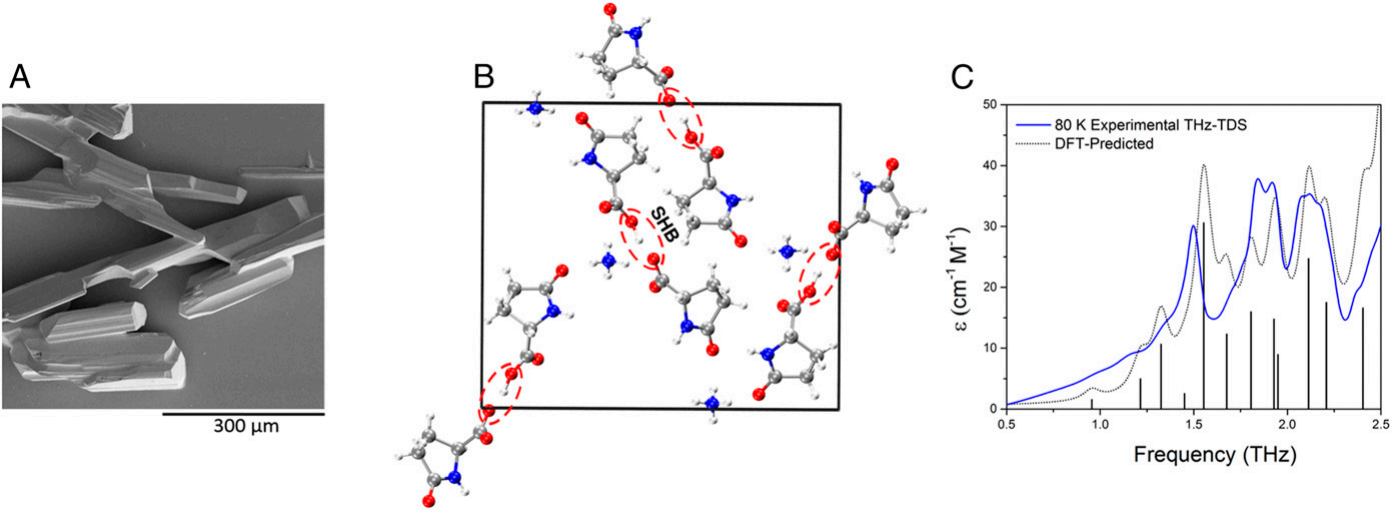
L-glu forms L-pyro-amm upon heating. (*A*) SEM image of crystals of L-pyro-amm dried. (*B*) XRD analysis of heated L-glu sample show the newly formed structure, L-pyro-amm. Geometry optimizations show that eight pyroglutamine groups in pairs, and four ammonium ions (144 atoms) for each dimer of pyroglutamine pairs, are complexed in the crystal. An SHB of 2.45 Å (within red dashed lines) is present near the ammonium ion (white: hydrogen, red: oxygen, blue: nitrogen, and gray: carbon). (*C*) Experimental (blue line) and theoretical (gray line) THz-TDS of the L-pyro-amm sample are in agreement and confirm the presence of the new L-pyro-amm structure.

We first investigated the structure of L-pyro-amm, which formed after incubation of L-glu for 8 d at 65 °C, using SEM and observed crystal structures shown in Fig. 1*A*. However, in order to investigate whether L-glu had indeed changed its crystal structure arrangement, we performed XRD analysis of the resulting material. In Fig. 1*B*, we show the crystal structure of the heated L-glu structure, which we termed L-pyro-amm, and the published crystal structures of L-glu and L-pyroglutamine (L-pyro) in *SI Appendix*, Fig. S1. Note, the L-pyro structure was analyzed, as it displayed structural similarities to the newly formed L-pyro-amm. Figures were obtained from geometry optimizations of the nuclear positions of the atoms using experimental densities. L-pyro-amm consists of eight pyroglutamine groups and four ammonium ions (144 atoms) complexed within the crystal (Fig. 1*B*). In contrast, as shown in *SI Appendix*, Fig. S1*A*, L-glu consists of four glutamine molecules (80 atoms) in the unit cell, which form HBs involving the termini and side chain. Furthermore, as shown in *SI Appendix*, Fig. S1*B*, L-pyro consists of 12 pyroglutamine molecules (192 atoms) in the unit cell forming HBs involving the NH and COOH groups.

L-pyro-amm has a rather unique HB network structure since four of the pyroglutamine molecules are deprotonated and hence have a nominal negative charge, while the other four molecules are neutral. One of the important implications of this difference is that L-pyro-amm contains a very SHB. The red circled regions in Fig. 1*B* correspond to an SHB with a length of 2.45 Å, while those in L-glu and L-pyro range between ∼2.55 to 2.85 Å (*SI Appendix*, Fig. S1).

The structural change was further confirmed using THz-TDS measurements, as this technique is strongly dependent on the bulk packing arrangement as well as on the internal covalent structure of the molecules (44). The THz-TDS spectrum of the resulting solid, as well as the solid-state DFT-predicted spectrum based on the SCXRD-determined structure, is shown in Fig. 1*C* (full spectral assignment available in *SI Appendix*, Fig. S2). The agreement between the experimental and theoretical spectra further supports that full conversion of the sample occurs and therefore enables additional investigations into the structural and electronic properties of the material. The agreement is also indicative of the ability of the theoretical model to not only reproduce the experimental structure but also the weak forces found in solid structures.

**Fig. 2.**
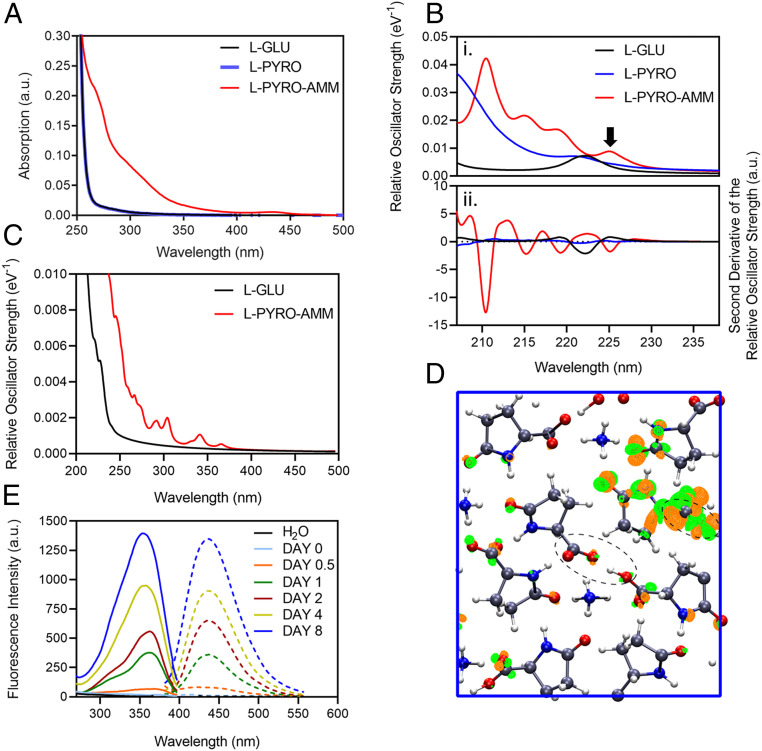
Optical properties of L-pyro-amm are distinct from L-glu and L-pyro. (*A*) Absorption spectra of 0.3 M L-glu (black), L-pyro (blue), and L-pyro-amm (red) (L-glu incubated for 8 d at 65 °C) in water taken between 200 to 500 nm shows primarily features of L-pyro-amm. (*B*) Absorption spectra of L-glu, L-pyro, and L-pyro-amm obtained from periodic DFT calculations with the B3LYP functional. L-pyro-amm features the lowest lying excited states, which are characterized by the largest oscillator strengths. (*C*) Absorption spectra for L-glu and L-pyro-amm obtained from periodic simulations at room temperature. The spectra were computed by averaging over 25 frames randomly sampled from the AIMD simulations. (*D*) The excited-state electron density computed for L-pyro-amm from the optimized structure computed at the first peak (arrow in *B*). The lowest excited-state density shows a response from various parts of the crystal structure including the pyroglutamic acid ring and the SHB region (see dashed circle). The orange and green surfaces correspond to regions involving a decrease and increase in electron density, respectively, shown at an iso-value of 1 × 10^−5^. (*E*) A total of 1 M L-glu was incubated at 65 °C, and the excitation and emission spectra were measured over time. Excitation spectra were measured between 250 to 400 nm with the emission set at 420 nm, and emission spectra were measured between 380 to 560 nm with the excitation set at 360 nm.

We first investigated whether there were any differences in the optical properties associated with the three crystal structures. Comparing the absorption of L-glu, L-pyro, and L-pyro-amm in water, we show that only L-pyro-amm has a significantly red-shifted absorption, which lies in the 275 to 320 nm range, whereas both L-glu and L-pyro primarily absorb in the deep ultraviolet (UV) (<250 nm) (see Fig. 2*A* for details).

We next compared the experimental absorption spectra of L-glu, L-pyro, and L-pyro-amm with the ones obtained from TDDFT. We highlight here that the small size of the systems permitted us to determine the spectra using a hybrid functional, thereby not only advancing the quality of our theoretical predictions from previous studies (5, 42, 43) but also coupling the optical properties directly to different vibrational modes.

Fig. 2*B* illustrates the absorption spectra obtained for the TDDFT calculations on the three periodic systems in the ground state (i.e., at 0 K). Fig. 2 *B*, *i* shows the relative oscillator strength as a function of the frequency, while Fig. 2 *B*, *ii* illustrates the second derivative of the oscillator strength permitting the positions of the maxima in the spectra to be more easily identified. The spectra reveal some striking differences between the different systems. Interestingly, we observe that L-pyro is essentially dark throughout the frequency range up to ∼6 eV. On the other hand, L-pyro-amm shows the presence of more structure in the spectrum. Specifically, it is the only system for which the spectrum features a low-energy excitation at 226 nm (5.5 eV) and subsequently other peaks slightly above 220 nm (5.625 eV) and 216 nm (5.75 eV). While L-glu exhibits a peak at 222 nm (5.58 eV), it is dark up to 206 nm (∼6 eV).

We have previously shown that thermal fluctuations and in particular nuclear vibrations, such as proton transfer, have a large impact on the absorption spectra of peptide structures as compared with the 0 K behavior (5, 45–47). In Fig. 2*C*, we show that, compared with the 0 K spectra in Fig. 2*B*, thermal fluctuations cause a large red shift to around 3.4 eV (365 nm) for L-pyro-amm, close to what is observed experimentally. These spectra were computed averaging over 25 frames sampled from the molecular dynamics simulations. Interestingly, no such effect is observed for L-glu, which remains weakly absorbing up to more than 5 eV (247 nm) as seen at 0 K.

In order to understand better the physical origin of the low-energy excitation at 226 nm (∼5.5 eV) in L-pyro-amm, we computed the electron response density at this frequency. This is illustrated in Fig. 2*D*, where we observe that most of the electron response is localized in regions around the pyroglutamine rings as well as regions near the SHB (see dashed circle in Fig. 2*D*). The optical response thus entails a charge reorganization involving several parts of the molecular crystal. It is interesting to note here that since L-pyro contains the same pyroglutamine rings as L-pyro-amm but absorbs more in the UV spectral rather than in the visible region, as shown experimentally, we conclude that the structural changes in the crystal in the presence of the SHB, which distinguishes L-pyro from L-pyro-amm, are responsible for the fluorescence and the relatively large Stokes shift observed in L-pyro-amm as shown experimentally in Fig. 2*E*.

We next investigated whether the above structures also display fluorescence excitation and emission properties as has been observed for amyloid-like structures reported previously (5, 42, 43). Fig. 2*E* shows the excitation scan from 250 to 400 nm (solid lines) with the emission set at 430 nm of L-glu in water at day 0 to 8 after incubation at 65 °C. We observe an excitation peak at around 360 nm, which is similar to what we have measured previously for amyloid proteins (5). The corresponding emission scan (dashed lines) with the excitation set at 360 nm and emission from 380 to 560 nm showed an emission peak around 430 nm, again lying in the same visible range as for amyloid fibrils. When the L-pyro-amm solution was dried, the excitation and emission peaks were slightly blue shifted (*SI Appendix*, Fig. S3*A*), which may be due to a change in the molecular environment in the dried state. Importantly, we do not see any fluorescence in L-glu (without heating, i.e., at day 0; Fig. 2*E*.). To determine the importance of the ammonium ion experimentally, L-pyro was incubated in water and heated at 65 °C for 8 d, and only a very weak fluorescence has been detected (*SI Appendix*, Fig. S3*B*).

**Fig. 3.**
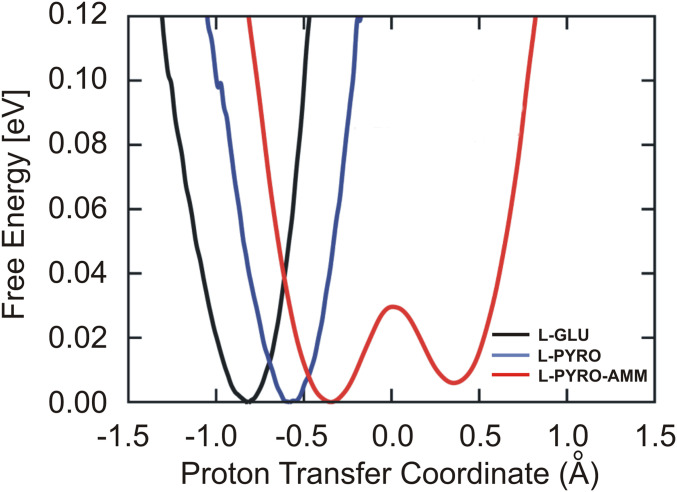
Free energy profiles along the proton transfer coordinate show only L-pyro-amm displays a double-well potential. Free energy profiles along the proton transfer coordinate are displayed for L-glu (black), L-pyro (blue), and L-pyro-amm (red) at RT. The L-glu and L-pyro display single-well potentials, while the L-pyro-amm system is the only one the exhibits a double-well potential, implying that there is proton transfer from one side to the other.

As alluded to earlier, one of the factors that distinguishes L-pyro-amm from the other systems is the presence of the SHB (highlighted by red circles in Fig. 1*B*) and the presence of the ammonium ion. In order to characterize the behavior of the SHB, we conducted AIMD simulations of the three systems at 300 K and examined the proton transfer coordinates defined as the difference in distance between the proton (H) and the two oxygen atoms (O1 and O2) that sandwich it. This is commonly referred to as the proton transfer coordinate (d_O1-H_-d_O2-H_) as shown in Fig. 3 for different types of HBs in the crystals. It is clear that the SHB in L-pyro-amm is characterized by a double-well potential. The barrier associated with this proton transfer is on the order of thermal energy, indicating that zero-point energy would make the proton transfer barrierless (48). An examination of similar proton transfer coordinates for HBs in L-glu and L-pyro show that they are characterized by only single-well potentials.

The presence of the double-well potential in the system with the SHB is consistent with numerous previous studies investigating the role of pressure, for example, on the evolution of HBs in crystals (49–52) as well as in proton transfer processes in liquid water where HB fluctuations to shorter distances (53, 54), like those observed in the L-pyro-amm system, lead to a change in the proton transfer potential.

**Fig. 4.**
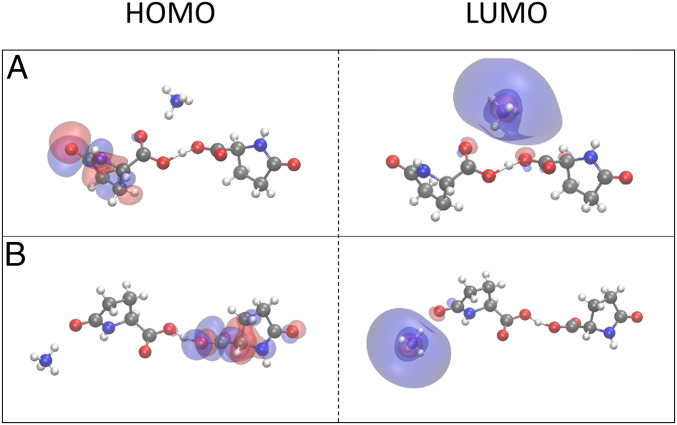
Comparison of the HOMO and LUMO orbitals on two L-pyro-amm models. L-pyro-amm structures are presented where the ammonium cation is located directly near the HB (*A*) and where the ammonium cation is located away from the HB (*B*). While both models predicted charge-transfer HOMO–LUMO states, only in the case of *A* is the transition predicted to be in the vicinity of the experimentally observed peak, 304 nm compared with 669 nm for *B*.

The nature of the optical properties is sensitive to the environment in which the glutamine molecules reside. It has previously been reported that charged amino acids already display an absorption in the range of 250 to 350 nm that is significantly red shifted (55, 56). The origins of the low-energy absorption were attributed to charge-transfer excitations. The simulations of these systems were performed in the gas phase rather than considering the protein environment such as shown for L-pyro-amm in Fig. 2*D*. In comparison with the results presented in Fig. 2*D*, data presented in Fig. 4 show that the origins of the electronic transitions equally arise from a charge transfer between the highest occupied molecular orbital (HOMO) on the anionic dimer, and the lowest unoccupied molecular orbital (LUMO) centered on the ammonium cation when performed in the gas phase. Interestingly, the correct transition energy is only predicted when the ammonium cation is spatially near the center of the dimer, which corresponds to the delocalization of the negative charge and the SHB. Two generalized geometries, with the ammonium cation near the SHB (as seen in Fig. 4*A*) and away from the SHB (Fig. 4*B*), with the corresponding HOMO and LUMO orbitals are shown. The results predict a transition of 304 nm for the dimer (*A*) and 669 nm for the dimer (*B*), with the dimer (*A*) most closely resembling the chemical environment present within the crystalline material.

The results show that charge transfer is capable to lead to absorption in the near ultraviolet when investigated in the gas phase, that is, neglecting the direct protein environment. However, including the protein environment in the molecular crystal (Fig. 2*D*) results instead in the excitation being a charge reorganization involving several different molecular groups of the crystal. Indeed, by shuffling the protons along the SHBs in the ground state, we observe an electronic response involving the entire structural units of L-pyro-amm, including both the hydrogen-bonded regions and the pyroglutamic acid rings when the protein environment is accounted for (*SI Appendix*, Fig. S4).

**Fig. 5.**
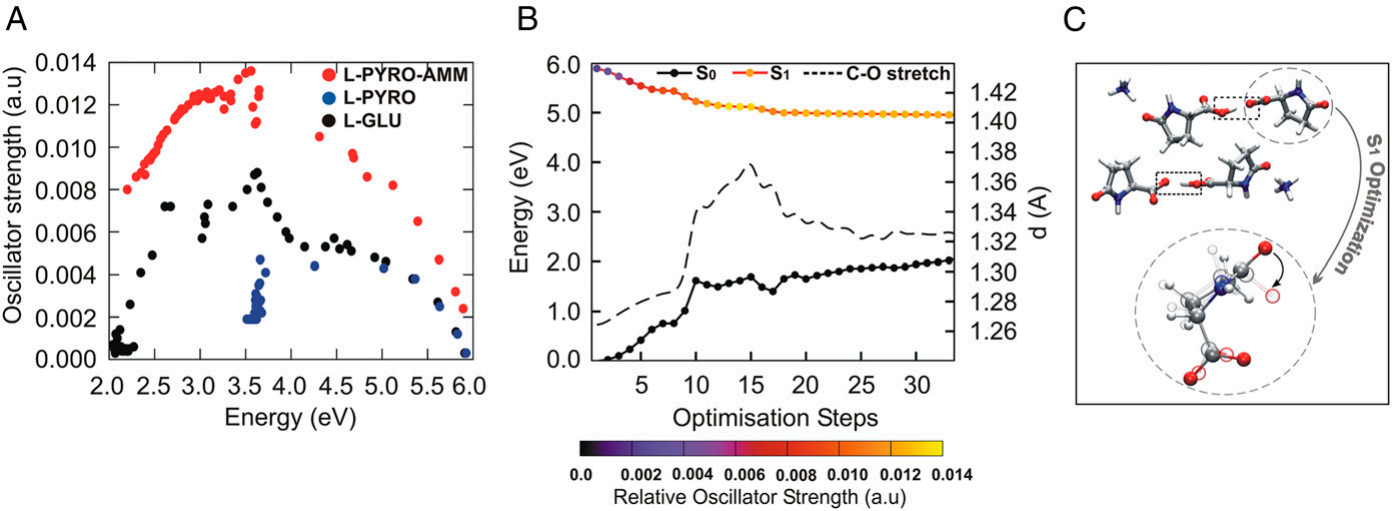
L-pyro-amm optical properties as seen through excited state optimizations. (*A*) Scatter plot of the oscillator strengths versus the emission energy (defined as the difference between the first excited state and the ground state) during the excited optimizations of L-glu (black), L-pyro (blue), and L-pyro-amm (red). (*B*) Ground- and excited-state energies are plotted as a function of the excited-state optimization of the system shown in *C*. The curves on the excited state are color coded with the oscillator strengths. S_0_ and S_1_ refer to the ground- and excited-state energies, respectively. (*C*) A snapshot of the optimized excited-state cluster also showing the lengthening of the carbonyl bond and the deplanarization of the ring.

Up to this point, we have shown that proton transfer along SHBs is an important part of the structural fluctuations in the ground-state structure of L-pyro-amm. In order to characterize the nuclear relaxation upon photoexcitation, we conducted both excited-state optimizations as well as excited-state molecular dynamics simulations. We first show the results from the excited-state optimizations obtained from clusters carved out from the different glutamine crystals and surrounded by a continuum dielectric constant of 80. Fig. 5*A* shows a scatter plot of the difference between the first excited-state and ground-state energies as well as the corresponding oscillator strengths for the three systems, L-glu, L-pyro, and L-pyro-amm. The scatter plots were obtained over the course of the excited-state optimization. We observe that the L-pyro-amm system is characterized by the largest oscillator strengths peaking at ∼3.5 eV (354 nm), which is consistent with our experimental findings. Although L-glu and L-pyro also have a peak at around 3.5 eV, it is much weaker than the one of L-pyro-amm. We thus decided to focus on a series of excited-state optimizations for various clusters of L-pyro-amm.

Fig. 5*B* shows the evolution of the first excited-state energy for one of the L-pyro-amm clusters (Fig. 5*C*) across the optimization steps. We find that the excited-state energy drops by 0.5 eV combined with an enhancement of the oscillator strength, while the ground-state energy, shown in solid black, rises by about 1.5 eV. In this case, the closing of the energy gap to ∼3 eV is associated with an increase of the carbonyl oxygen bond, as observed in previous studies (49–51), as well as a deplanarization of the pyroglutamine ring (Fig. 5*C*). Similar features are also observed in other clusters, including one with the ammonium ion closer to the short HB (*SI Appendix*, Fig. S5).

**Fig. 6.**
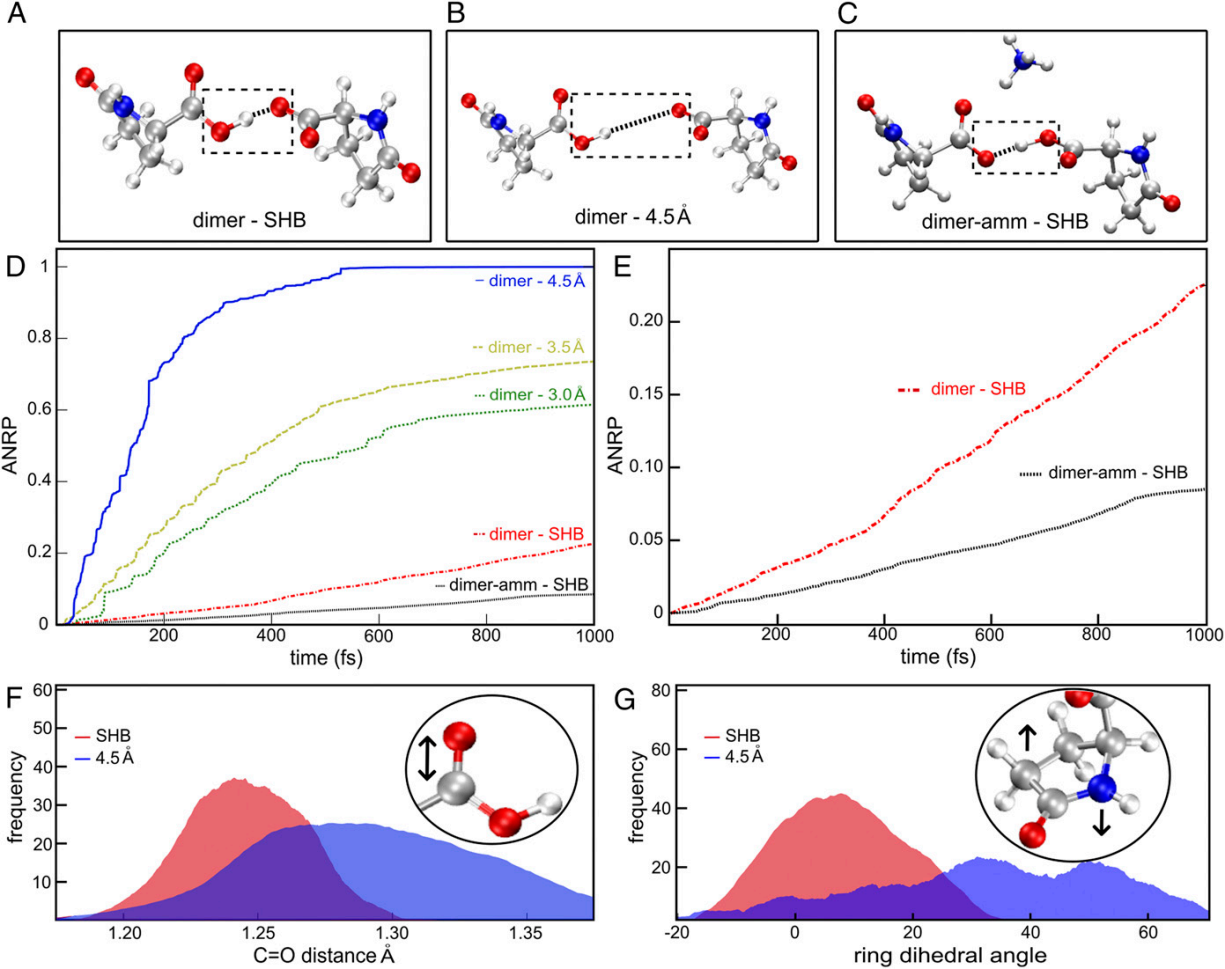
Excited-state AIMD highlights how the presence of an SHB and the ammonium ion is enhancing fluorescence. Excited-state AIMD performed for the (*A*) L-pyro dimer, (*B*) the L-pyro dimer constraining the SHB distance, and (*C*) L-pyro-amm is shown. (*D*) Accumulated nonradiative S_1_ → S_0_ decay probability, ANRP(t), for the L-pyro dimer (dimer-SHB) and the constrained SHB L-pyro (dimer-), where the HB distance was fixed at 3.0, 3.5, and 4.5 Å, and for the L-pyro-amm dimer (dimer-amm-SHB). (*E*) Accumulated nonradiative decay probability for L-pyro (red discontinuous line) and L-pyro-amm (black dashed line). (*F*) Carboxyl C = O distance histogram for the L-pyro-amm dimer at the SHB distance (red curve) or constraining the HB to 4.5 Å (blue curve). (*G*) Ring dihedral angle histogram for the L-pyro-amm dimer at the SHB distance (red curve) or constraining the HB to 4.5 Å (blue curve).

To further explore the preceding mechanisms, we next turn to deploying nonadiabatic decay estimations using excited-state AIMD simulations as implemented in the LIO quantum-chemical package (https://github.com/MALBECC/lio) (31–34). This approach enables investigating the S1 → S0 de-excitation probability, providing a clear interpretation of the ensuing optical properties of L-pyro-amm (see *Materials and Methods* for details). The nature of the S1 → S0 transition is mainly a *v*’’ = 0 ← *v*’ = 0 (where *v*’ and *v*’’ are the vibrational quantum numbers in the electronic ground and excited state, respectively) with a Franck–Condon (FC) factor between ground vibrational levels of ∼0.82 (*SI Appendix*, Fig. S6), and therefore the excited-state simulation has been initiated by a vertical excitation to the S1 state. We performed two sets of excited-state simulations: 1) varying the strength of the SHB to study its influence on the fluorescence of the L-pyro dimer (Fig. 6 *A* and *B*) and 2) comparing L-pyro with L-pyro-amm dimers to assess the role of the ammonium ion on the transient excited-state dynamics (Fig. 6*C*). Each trajectory was propagated for 1 ps from which the nonradiative transition probability, $NRP_{S_{1}\to S_{0}}\left(t\right)$, was determined by the fewest switches trajectory surface hopping approach (35–37). Fig. 6*D* shows the time evolution of the accumulated nonradiative decay probability, ANRP(t), for various HB lengths (*Materials and Methods*). The ANRP(t) at time t represents the total probability for S_1_ → S_0_ nonradiative relaxation in the interval {0,t}. A large ANRP(t)value implies a high likelihood of decaying nonradiatively to the ground state. On the other hand, a lower ANRP(t)would involve a longer excited-state lifetime increasing the fluorescence probability. Fig. 6*D* demonstrates unequivocally that the presence of the SHB significantly reduces the chances of nonradiative relaxation toward the ground state. Examining the energy gap between the excited and ground state for this system shows that it occurs at ∼3 eV (413 nm) (*SI Appendix*, Fig. S7*A*), consistent with fluorescence in the blue/green visible regime.

Furthermore, the presence of the ammonium ion not only increases the oscillator strength, as observed in Fig. 5*A*, but it also enhances, albeit by a subtle amount, the probability of trapping the system in the excited state. Collectively therefore, Figs. 5 and 6 show that the ammonium ion can enhance the fluorescence yield in two ways: first, by increasing the fluorescent rate constant and second, by reducing the nonradiative decay probability.

The excited-state optimizations shown in Fig. 5 illustrate the important role played by the vibrational distortions in the excited state on the ensuing optical property. In agreement with this, Fig. 6 *F* and *G* shows the distribution of the C = O bond length and the ring deplanarization (computed as the sum of its internal dihedral angles) for the dimer systems with the SHB and weaker (longer) HBs *Left* and *Right*, respectively. Interestingly, we see that the trajectories that undergo nonradiative decay are characterized by both a significant increase in the carbonyl stretch (C = O bond length) by about a tenth of an Angstrom and a large deplanarization of the dihedral angle. The combined distortion of these vibrational coordinates leads to nonradiative decay as observed in the dimer system with longer HBs. In the system with the SHBs, these modes are hampered, which essentially prevents the system from easily accessing conical intersections. This increases the likelihood of observing fluorescence in the L-pyro-amm structure characterized by short HBs. Therefore, both our experiments and simulations demonstrate the crucial role played by the SHB in determining the fluorescence properties of L-pyro-amm.

## Conclusions

The experimental and theoretical findings presented here elucidate a rather complex molecular mechanism associated with the nonaromatic intrinsic fluorescence in protein-like structures. In the case of L-glu, a chemical reaction creates a newly formed structure involving a cyclized pyroglutamic acid ring. This new structure features absorption in the ultraviolet and emission in the visible range, very similar to the chemically distinct amyloid fibrils (4, 5, 11, 12, 57–59). The structural chromophore responsible for the optical properties in this new protein-related structure arises from an HB network associated with structures involving SHBs. Indeed, we have shown previously that, similar to L-pyro-amm, the crystallized structure of the hydrophobic core of amyloid-β (Protein Data Bank 2Y3J), L-pyro-amm, contains an SHB (60) leading to a double-well potential in the ground state. The presence of SHBs along which proton transfer occurs as well as specific ionic interactions in close proximity, such as involving the ammonium ion of L-pyro-amm, affect the optical properties of the protein-like structure. Although the fluorescence observed in these systems is much weaker compared with conventional fluorophores, the physical and chemical properties of the HB network reported here may be a generic feature across many other peptide structures.

Our nonadiabatic molecular dynamics simulations demonstrate that the presence of SHBs together with specific environmental conditions hinder vibrational deformations that can access conical intersections as previously speculated (5). As we have seen, the SHBs are permissive to proton transfer leading to double-well potentials (49–54). Specifically, a double-well proton transfer potential may prevent nonradiative decay mechanisms in two possible ways. Firstly, as we proposed in an earlier work, it may rise the barriers for accessing conical intersections on the excited state and thereby increasing the chances of fluorescence. Secondly, the SHBs also stiffen the HB network and reduce the fluctuations of modes associated with the carbonyl groups, which, if activated, could lead to nonradiative decay. The role of carbonyl groups is also consistent with recent theoretical and experimental studies showing their importance for nonaromatic fluorescence (61, 62).

We conclude by remarking that SHBs have recently been observed in different biological systems which have long been associated with either intrinsic fluorescence, such as nicotinamide adenine dinucleotide phosphate (NADP/NAD) (63) and flavin adenine dinucleotide/flavin mononucleotide (FAD/FMN) (64), the light-sensing chromophore in photoactive yellow protein (65), or in the active site of many enzymes, such as hydrolases and oxidoreductases (66, 67), many of which consist of highly complex HB structures similar to amyloid proteins. Furthermore, it has been recently reported that one in every 16 HBs in over 1,600 proteins is characterized by a SHB (67). Thus, the mechanisms we espouse here may be more general. Our findings offer the possibility of designing novel biomaterial for applications in optical sensing or the design of novel biocompatible catalysts.

## Supplementary Material

Supplementary File

## Data Availability

Excel spreadsheet data have been deposited in the University of Cambridge Repository (https://doi.org/10.17863/CAM.57945). SCXRD data are available at the Cambridge Crystallographic Data Centre (https://www.ccdc.cam.ac.uk/), CCDC No. 1981551.
