# Supplementary material for: Short hydrogen bonds enhance nonaromatic protein-related fluorescence
Source: Proc Natl Acad Sci U S A. 2021 May 17;118(21):e2020389118. doi: 10.1073/pnas.2020389118 (PMC8166056; doi:10.1073/pnas.2020389118)
Supplement: Supplementary File [file pnas.2020389118.sapp.pdf]

PNAS
www.pnas.org

Supplementary Information for
Short hydrogen bonds enhance non-aromatic protein-related fluorescence.

Amberley D. Stephens, Muhammad Nawaz Qaisrani, Michael T. Ruggiero, Gonzalo Diaz Miron, Uriel N. Morzan, Mariano C. González Lebrero, Saul T.E. Jones, Emiliano Poli, Andrew D. Bond, Philippa J. Woodhams, Elyse M. Kleist, Luca Grisanti, Ralph Gebauer, J. Axel Zeitler, Dan Credgington, Ali Hassanali, Gabriele S. Kaminski Schierle

Ali Hassanali, Gabriele S. Kaminski Schierle
Email: ahassana@ictp.it, gsk20@cam.ac.uk

This PDF file includes:

Figures S1 to S7

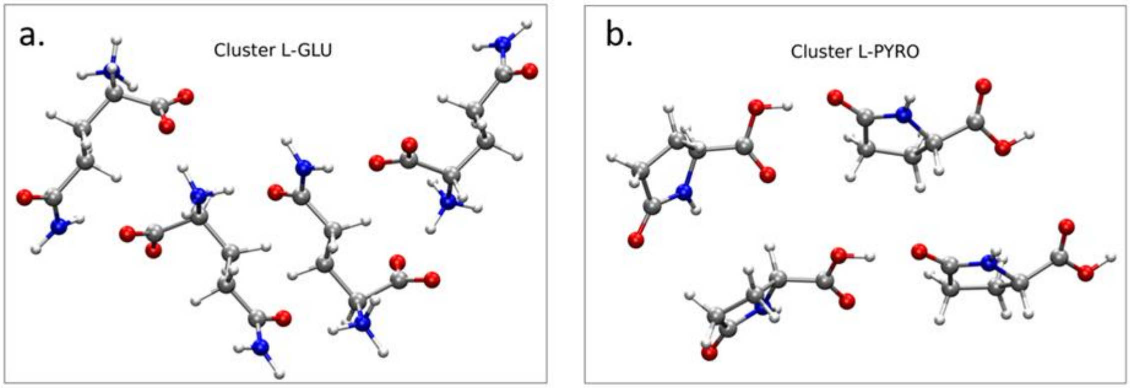

Fig. S1. Clusters of L-glu and L-pyro. Structural representations of (a) L-glutamine (L-glu) and (b) L-pyroglutamine (L-pyro) clusters from the published crystal structure**.**

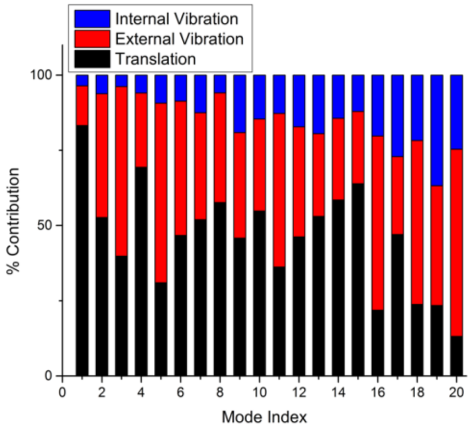

Mode Number	Frequency (THz)	Intensity (km mol^-1)	Assignment
1	0.955	0.22	Antisymmetric translation in b
2	1.215	0.71	Coupled translation in a and rotation about bc
3	1.325	1.52	external rotation about b
4	1.450	0.36	Asymmetric translation in c
5	1.554	4.37	External rotation about a
6	1.676	1.76	Translation in a and external asymmetric rotation about H-bond coordinate
7	1.805	2.28	External asymmetric rotation of dimer pairs
8	1.929	2.11	External symmetric rotation of dimer pairs
9	1.950	1.28	Asymmetric rotation about h-bond coordinate, ammonium translational motion
10	2.113	3.53	Translation and rotation (breathing) around ammonium cation
11	2.208	2.5	Symmetric external rotation of entire formula units
12	2.404	2.37	In phase external rotation perpendicular to h-bond coordinate
13	2.502	0.45	Out of phase external rotataion perpendicular to h-bond coordinate
14	2.564	0.73	External out of phase rotataion of h-bonded chains about b
15	2.565	8.91	Out of phase external rotation of individual pyroglutamic molecules with translation of ammonium cation
16	2.654	18.09	External vibration coupled with torsion of the COOH group
17	2.679	0.29	External rotation and torision of pyroglutamic ring
18	2.776	4.94	External rotation and torsion of ring and carboxyl group
19	2.988	2.68	External rotataion and translation of ammonium

Fig. S2. Full spectral assignment of THz data. The top chart shows the contribution to each IR-active mode including external translations and hindered rotations, and internal vibrational motions (i.e. torsions), while the bottom table lists the detailed assignment for each mode.

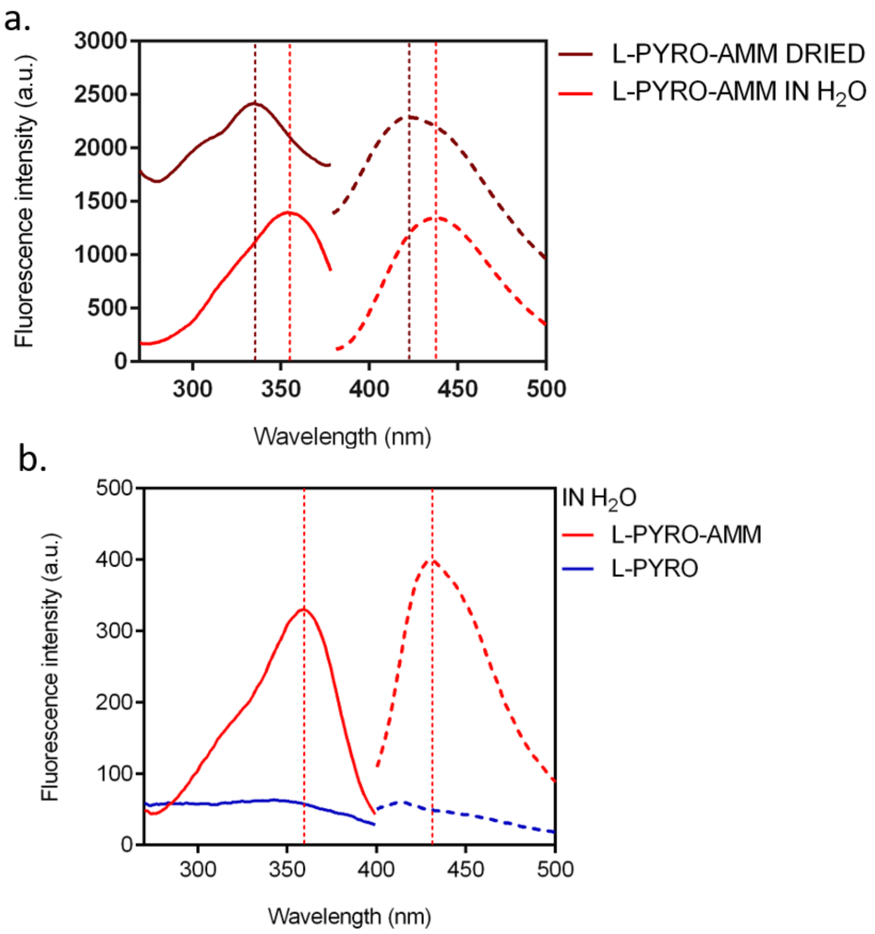

Fig. S3. L-pyro-amm has blue shifted fluorescence when dried and displays higher fluorescence intensity than L-pyro. (a)The excitation peak of L-pyro-amm when dried (solid dark red line) is blue shifted with a peak maximum ~ 340 nm compared to L-pyro-amm in H2O (solid red line) which has a peak maximum ~ 360 nm. The emission peak of L-pyro-amm when dried (dashed dark red line) is also blue shifted, with a peak maximum ~ 420 nm, compared to L-pyro-amm in H2O (dashed red line) with a peak maximum ~ 430 nm. (b) 1 M L-glu and 1 M L-pyro (blue) were incubated in H2O for 8 days at 65°C. After 9 days, the L-glu had converted into the L-pyro-amm structure (red). L-pyro-amm has a clear excitation peak maximum at ~360 nm and emission peak maximum at ~ 430 nm, while L-pyro (blue), although not completely dark, has no clear excitation or emission peak.

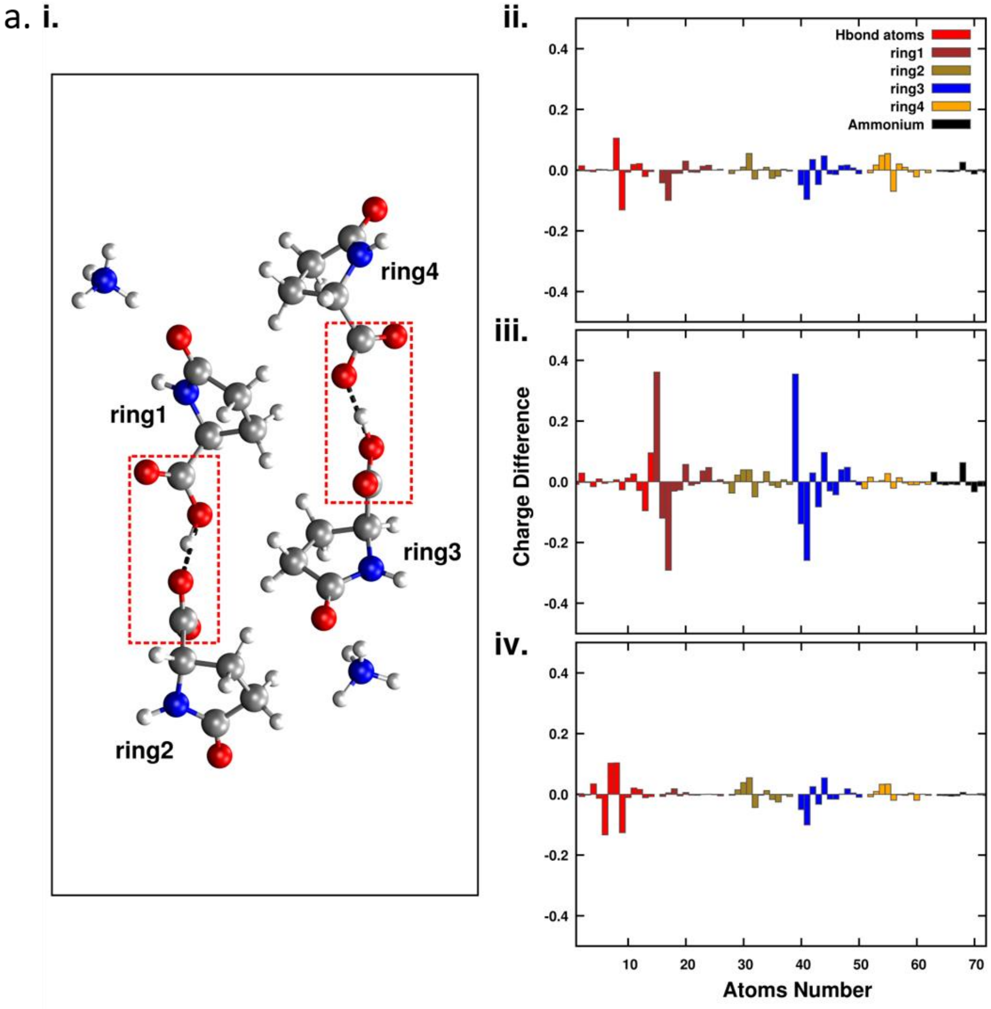

Fig. S4. The optical properties of L-pyro-amm are sensitive to the environment and involves the electronic response of the entire structure. (a)(i) L-pyro-amm cluster used to examine the sensitivity of the optical response on different parts of the cluster upon moving different protons. (ii) Charge differences between the ground and excited state are computed using restrained electrostatic potential atomic partial charges (RESP) for the cluster shown in i). Note, the electronic response involves all the atoms of the cluster. (iii) The two protons in the rectangle regions are displaced to be in the centre of the hydrogen bond and the charge differences are then computed. As illustrated, the proton displacement leads to a larger change in magnitude of the charges. (iv) The charge differences are computed for another nuclear configuration for which the protons are kept fixed but the O—O distance is increased from 2.45 to 3.2 Angstroms. The charge differences obtained here are quite similar to the original condition shown in (ii).

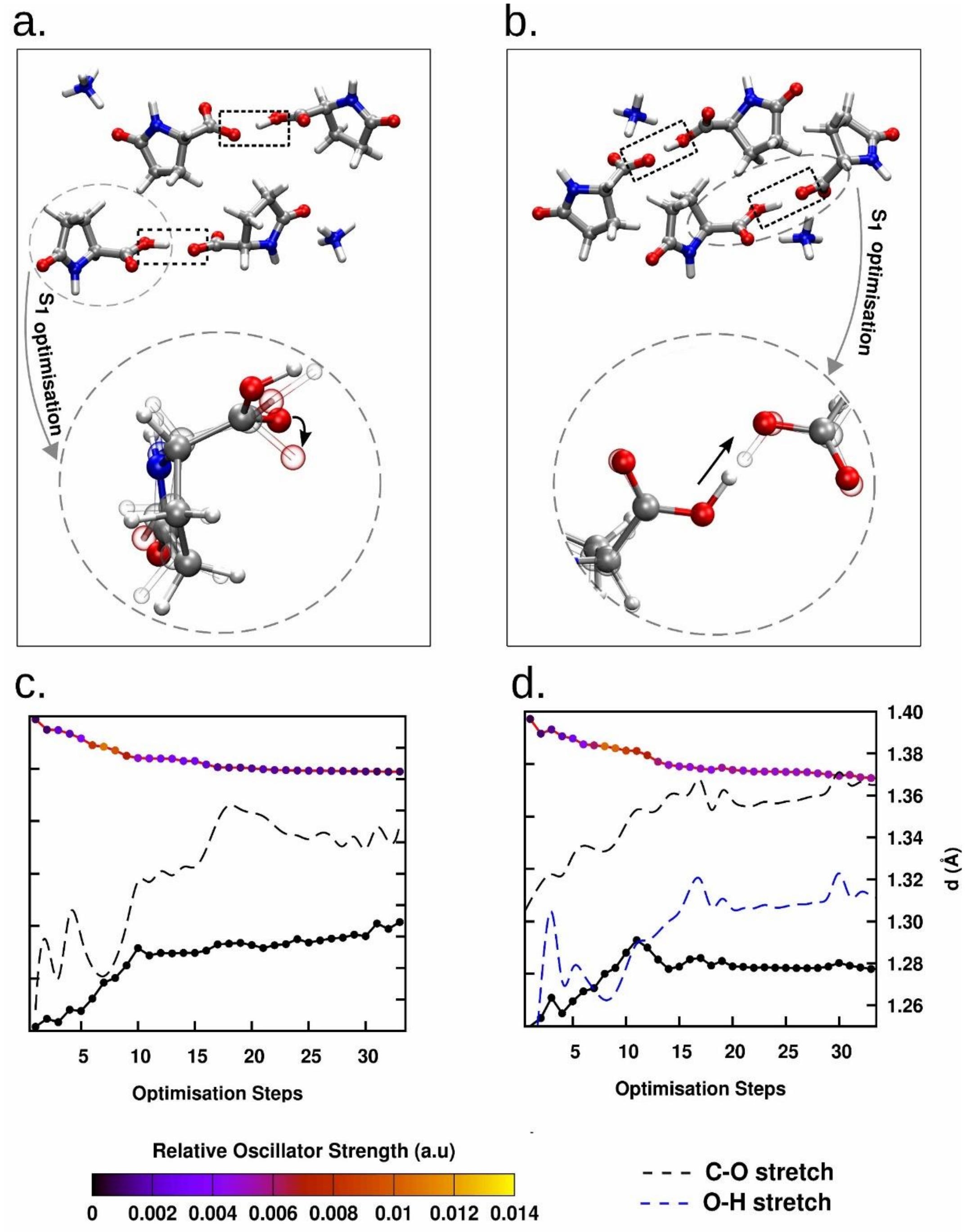

Fig. S5. L-pyro-amm undergoes vibrational distortions upon excitation as seen through excited state optimisations. (a-b) correspond to two additional clusters to the one reported in the main text that were built from the L-pyro-mm crystal structure. c) and (d) correspond to the evolution of the excited state energy and ground-state energy over the course of the optimisation. Also shown, are the changes in the C-O bond lengths (dashed black) which increases on the excited state surface. In (d) we show the O-H bond stretch where a proton transfer is observed on the excited state.

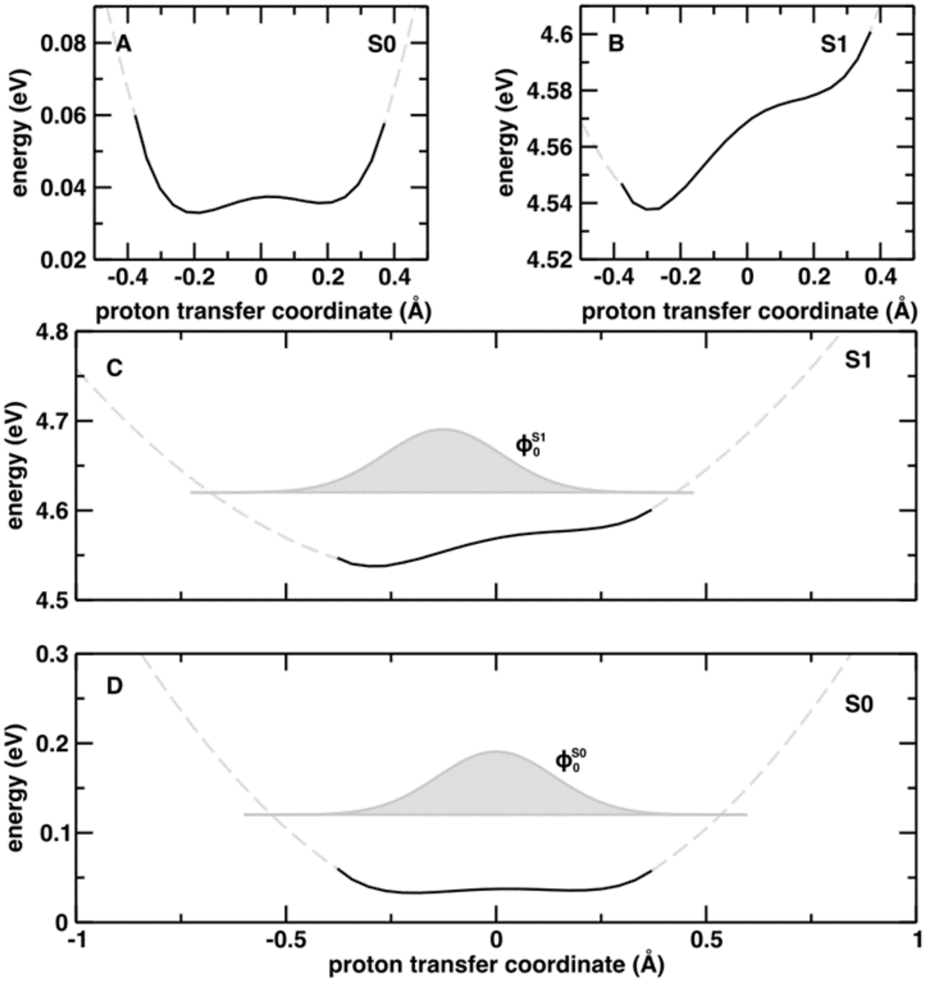

Fig. S6. The S0 and S1 nuclear wavefunctions associated to the SHB proton transfer coordinate reveal a vibronic transition dominated by a 0←0 character. The calculated potential energy surfaces (black continuous lines) are prolongated with a quadratic fitting (gray dashed lines). Panels C and D illustrate the ground-state vibrational wavefunction in the S0 and the S1 electronic states (Φ_0^S0 and Φ_0^S1respectively). The resulting Franck-Condon factor between Φ_0^S0 and Φ_0^S1 is 0.82, showing that the nature of the S1 ← S0 transition is mainly a v''=0 ← v'=0 (where v' and v'' are the vibrational quantum numbers in the electronic ground and excited state respectively).

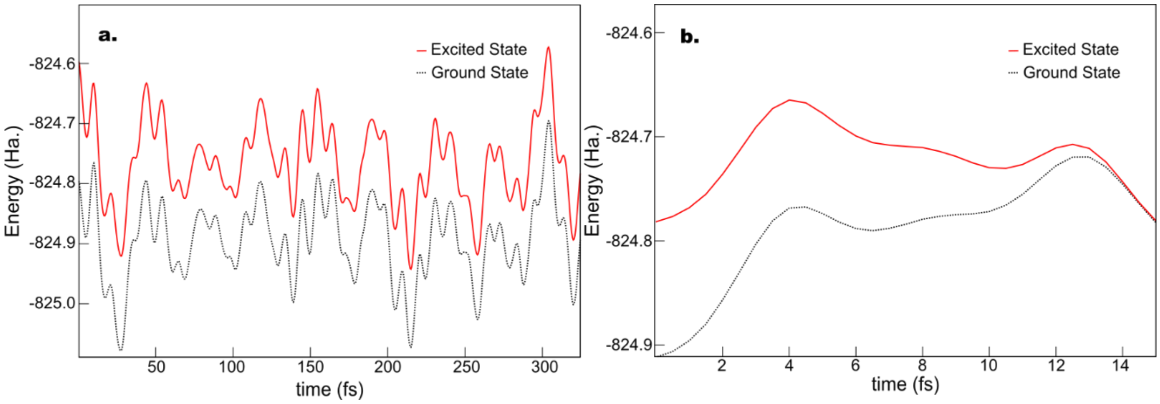

Fig. S7. The energy gap between excited and ground state of L-pyro-amm is consistent with the experiments and eventually deactivates via a conical intersection back to the ground state. (a) Shows the energy of the excited and ground state obtained from the excited state molecular dynamics simulations where non-adiabatic decay probabilities are estimated. We see clearly that the energy difference between the excited and ground state is approximately 3 eV (413 nm) consistent with experimental data. (b) Shows the same excited state and ground state energy for another simulation where the system de-activates very quickly within 10s of femtoseconds through a conical intersection where the ground and excited state energies intersect.
